# Association between physical activity and cardiovascular-kidney-metabolic syndrome in older Chinese adults: a nationwide, cross-sectional study

**DOI:** 10.7189/jogh.16.04149

**Published:** 2026-04-30

**Authors:** Qingmei Liu, Ji Shen, Yushan Zhang, Chi Zhang, Guoqing Fan, Lei Wang, Houguang Zhou, Jie Zhang, Hong Shi

**Affiliations:** 1 Department of Rehabilitation, Beijing Hospital, National Center for Gerontology; National Clinical Research Center for Gerontology; The Key Laboratory of Geriatrics of NHC; Institute of Geriatric Medicine, Chinese Academy of Medical Sciences, Beijing, China; 2 Department of Geriatrics, Beijing Hospital, National Center for Gerontology; National Clinical Research Center for Gerontology; The Key Laboratory of Geriatrics of NHC; Institute of Geriatric Medicine, Chinese Academy of Medical Sciences, Beijing, China; 3 Department of Basic Innovation Research, Beijing Hospital, National Center for Gerontology National Clinical Research Center for Gerontology; The Key Laboratory of Geriatrics of NHC; Beijing Key Laboratory of Aging Mechanism and Intervention Research on Aging-Related Diseases; Institute of Geriatric Medicine, Chinese Academy of Medical Sciences & Peking Union Medical College, Beijing, China; 4 Medical Center on Aging of Ruijin Hospital, Shanghai Jiao Tong University School of Medicine, Department of Geriatrics, Shanghai, China; 5 Huashan Hospital, National Clinical Research Center for Aging and Medicine, Fudan University, Department of Geriatrics, Shanghai, China

## Abstract

**Background:**

Cardiovascular-kidney-metabolic (CKM) syndrome represents a new framework to address the non-communicable disease burden in ageing populations. However, evidence on the association between physical activity and the integrated CKM syndrome is scarce, particularly among older Asian adults. We aimed to investigate this association in a large sample of older Chinese adults.

**Methods:**

This cross-sectional study utilised data from the China Ageing and Health Survey, including 41 829 community-dwelling adults aged ≥65 years. Physical activity was assessed using the validated Physical Activity Scale for the Elderly (PASE). Cardiovascular-kidney-metabolic syndrome was defined based on the 2023 American Heart Association criteria (Stages 1–4 *vs.* Stage 0). Multivariable logistic regression models were used to examine the association between PASE quartiles and CKM syndrome prevalence.

**Results:**

The prevalence of CKM syndrome was 80.3%. After full adjustment, a significant inverse dose-response relationship was observed between physical activity and CKM syndrome (*P* for trend <0.001). Participants in the highest physical activity quartile had 21% lower odds of CKM syndrome compared to the lowest quartile (adjusted odds ratio (aOR) = 0.79; 95% CI = 0.74–0.85). This inverse association was consistent across sexes but was strongly age-dependent: it was most pronounced among individuals aged ≥80 years (*P* for trend <0.001) and non-significant in the 65–69 age group (*P* for trend >0.05).

**Conclusions:**

Higher physical activity is independently associated with a lower prevalence of CKM syndrome in older Chinese adults. This inverse association was strikingly age-dependent and most pronounced in the oldest-old (aged ≥80 years). For aging populations globally, promoting accessible, age-appropriate physical activity may represent a high-priority, low-cost public health strategy to reduce CKM burden, particularly in this most vulnerable demographic.

The confluence of rapid population ageing and the rising prevalence of non-communicable diseases (NCDs) poses a substantial public health challenge globally, with nations like China at the forefront of this demographic and epidemiological transition [1]. This has spurred a paradigm shift from organ-centric disease management towards a more holistic, systems-based approach. A pivotal development in this area is the recent conceptualisation of cardiovascular-kidney-metabolic (CKM) syndrome by the American Heart Association [2]. Cardiovascular-kidney-metabolic syndrome provides an integrated framework that captures the progressive interplay between obesity, type 2 diabetes, chronic kidney disease, and cardiovascular disease, emphasising their shared pathophysiological pathways, such as insulin resistance and chronic inflammation [3,4].

While the CKM framework is a significant conceptual advance, its epidemiological profile and risk factors in diverse global populations, particularly within Asia, remain largely uncharacterised. China, with its vast and rapidly ageing population, represents a critical setting to investigate the burden and determinants of CKM syndrome [5,6]. Understanding the factors associated with CKM is essential for tailoring effective, large-scale prevention strategies in similar socio-demographic contexts.

Physical activity is a universally recognised cornerstone of healthy ageing and a powerful, non-pharmacological tool for NCD prevention [7,8]. Its benefits in improving glycaemic control, lowering blood pressure, and enhancing vascular function are well-established, directly counteracting the core mechanisms of CKM syndrome [9,10]. However, most evidence to date has focused on the association between physical activity and individual components of CKM, such as hypertension or diabetes, in isolation [11–13]. There is a paucity of research examining physical activity in relation to the novel, integrated CKM staging system, which is crucial for validating the syndrome’s clinical utility and promoting holistic lifestyle interventions.

This knowledge gap is particularly pronounced among older adults. In this demographic, physical activity is a multifaceted behaviour comprising not only structured exercise but also essential daily household and occupational tasks. Specialised instruments, such as the Physical Activity Scale for the Elderly (PASE), are designed to capture this broad spectrum of activity, offering a more realistic measure of daily energy expenditure than simple leisure-time activity logs [14]. To date, no studies have utilised such a comprehensive tool to explore the dose-response relationship between overall physical activity and CKM syndrome in a large, community-dwelling older adult population in China.

Therefore, this study leverages data from the China Ageing and Health Survey (CAHS) to address these critical gaps. Our primary objectives were to:

(1) investigate the association between physical activity levels, measured by the PASE, and the prevalence of CKM syndrome in a large sample of Chinese older adults;

(2) explore potential effect modification by age and sex, to identify high-risk subgroups and inform targeted public health strategies.

## METHODS

### Study population

**Figure 1:**
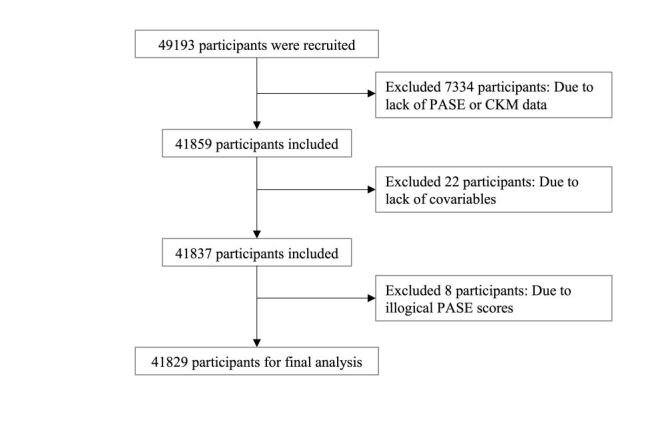
The flowchart of study population selection.

This study was based on data from the China Ageing and Health Survey (CAHS), a large-scale, nationwide survey assessing the health status of older adults in China. A sample of community-dwelling Chinese citizens aged 65 years and older was recruited via a multi-stage sampling method across diverse provinces. Local community health workers facilitated the recruitment process by approaching eligible older adults during household visits or at community health centres. The study protocol was approved by the Ethics Committee of Beijing Hospital (No. 2021BJYEC-114-01). Prior to data collection, all participants underwent a comprehensive explanation of the study's purpose and procedures. Written informed consent was subsequently obtained from all participants; for those with cognitive impairment or physical inability to sign, written consent was obtained from their legal representatives or designated proxies. Detailed descriptions of the survey design and data collection procedures have been published previously [15]. The participant selection process is detailed in **Figure 1**.

### PASE assessment

Physical activity was assessed using the Physical Activity Scale for the Elderly (PASE) [16], a validated instrument designed to measure physical activity levels among older adults over a one-week period. The PASE comprises three major domains:

(1) leisure-time activities (*e.g*. walking, sports, and recreational exercise),

(2) household activities (*e.g*. housework, gardening, and caring for others), and

(3) occupational activities (*e.g.* paid or volunteer work).

Each activity is assigned a weight based on its intensity and frequency, and a composite score is calculated to represent the overall level of physical activity. All assessments were conducted through face-to-face interviews by trained investigators.

### CKM syndrome assessment

The assessment of CKM syndrome was conducted based on the American Heart Association (AHA) Scientific Statement on Cardiovascular-Kidney-Metabolic (CKM) Syndrome (2023) [2]. To ensure the feasibility and consistency of assessment in a large population-based study, we adapted the AHA-recommended framework into a structured, five-stage classification (Stage 0–4), while maintaining alignment with the core conceptual domains of metabolic risk, disease progression, and clinical outcomes.

• Stage 0: No CKM health risk factors.

• Stage 1: Without the presence of other metabolic risk factors or CKD, but with overweight/obesity, abdominal obesity, or dysfunctional adipose tissue, defined as body mass index (BMI) ≥ 23 kg/m^2^.

• Stage 2: No established CVD or high predicted risk, but presence of metabolic risk factors (hypertension, type 2 diabetes, hypertriglyceridaemia) or chronic kidney disease.

• Stage 3: No established cardiovascular disease but at high cardiovascular risk, defined as a score of ≥4 on a simplified scale adapted from the WHO/ISH model based on age, systolic blood pressure, smoking status, diabetes, and total cholesterol [17].

• Stage 4: Clinically diagnosed cardiovascular, cerebrovascular, or kidney diseases, including heart failure, coronary heart disease, arrhythmia, cerebral haemorrhage, or cerebral infarction.

For the primary analyses, CKM syndrome status was dichotomised to indicate the presence (stages 1–4) or absence (stage 0) of CKM syndrome, resulting in a binary dependent variable (CKM = 0 for ‘no CKM syndrome’; CKM = 1 for ‘presence of CKM syndrome’).

All assessments were conducted through standardised, face-to-face interviews by trained investigators. This structured approach was designed to balance scientific rigour and operational feasibility, ensuring comparability with the AHA framework while enabling consistent classification across a large, community-based sample.

### Assessments of other covariables

Referring to previous studies [18,19], we selected variables that may potentially influence CKM syndrome as covariates, including sex, age, marital status, education, residential location (urban or rural), living alone, dietary habits (preference for salty food, sweet food, whole grains, and fruits/vegetables), and current alcohol consumption.

### Statistical analysis

All statistical analyses were performed using IBM SPSS Statistics 26.0 (IBM Corporation, Armonk, New York, USA) and *R*, version 4.5.1 (R Foundation for Statistical Computing, Vienna, Austria). Continuous variables were described as means and standard deviations (SD) or interquartile ranges (IQR), and categorical variables were presented as frequencies and percentages. Group comparisons across different CKM stages were conducted using analysis of the Kruskal-Wallis test for continuous variables and the χ^2^ test for categorical variables. Regarding missing data, participants with incomplete information on the PASE score and CKM components, or key covariates were excluded from the analytical sample using complete case analysis. The binary logistic regression models were used to examine the association between PASE and the presence of CKM syndrome. Three models were constructed sequentially, model 1 (unadjusted): included only the PASE as the independent variable; model 2 adjusted for demographic factors, model 3 additionally adjusted for dietary preferences, and current alcohol consumption. Smoking status was not included as a separate covariate in the primary multivariable models because it was already incorporated as a component of the CKM Stage 3 definition; its simultaneous inclusion as an independent covariate would constitute over-adjustment for that outcome category. Subgroup analyses were conducted to explore potential effect modification by sex (male/female) and age groups (65 to 69, 70 to 79, ≥80 years). To formally test whether age group modified the PASE-CKM association, a multiplicative interaction term (PASE × age group) was added to the fully adjusted model and evaluated by likelihood ratio test. A *P* < 0.05 was considered statistically significant.

Finally, several sensitivity analyses were conducted to further evaluate the robustness and potential nonlinear dose-response relationship of our findings. First, because the proportional-odds assumption was violated, a supplementary multinomial logistic regression was conducted with CKM stage (0–4) as the outcome and Stage 0 as the reference. Second, a sensitivity analysis additionally adjusting for smoking status was conducted to address potential residual confounding. Third, to evaluate the robustness of the Stage 1 definition, the primary analysis was repeated redefining Stage 1 using BMI≥24 kg/m^2^ and ≥28 kg/m^2^. Fourth, to minimise the potential for reverse causation and account for outlier effects, we re-estimated the fully adjusted models after excluding participants with a PASE score below the 10th percentile, those aged over 100 years, and those classified as CKM Stage 4. Lastly, to assess potential nonlinearity, restricted cubic spline (RCS) regression was performed with five knots placed at the 10th, 25th, 50th, 75th, and 90th percentiles of PASE; the 10th percentile served as the reference, and departure from linearity was evaluated via a likelihood ratio test.

## RESULTS

### Characteristics of Participants

A total of 41 829 older adults were included in the analysis, with a median age of 72.0 years (IQR = 69.0–78.0) and 52.5% being women. Among them, 80.3% met the criteria for CKM syndrome, with the distribution of CKM stages 0 to 4 being 19.7, 20.1, 28.5, 13.6, and 18.1%, respectively. Participants with CKM syndrome were generally older, and some differences were observed in sociodemographic characteristics (*P* < 0.001). In terms of lifestyle factors, CKM participants reported a greater preference for salty foods and lower consumption of whole grains, a higher prevalence of current alcohol consumption. Additionally, participants with CKM syndrome had significantly lower PASE scores (**Table 1**).

**Table 1:** Characteristics of participants

Characters	Total (n = 41 829)	Non-CKM (n = 8231)	CKM (n = 33 598)	*P-*value*
Age in years (IQR)	72.0 (69.0–78.0)	72.0 (68.0–77.0)	73.0 (69.0–78.0)	<0.001†
Gender (%)				0.724
*Female*	21 952 (52.48)	4334 (52.65)	17 618 (52.44)	
*Male*	19 877 (47.52)	3897 (47.35)	15 980 (47.56)	
Marital status (%)				<0.001†
*Never married*	569 (1.36)	150 (1.82)	419 (1.25)	
*Married*	33 746 (80.68)	6769 (82.24)	26 977 (80.29)	
*Widowed/divorced*	7514 (17.96)	1312 (15.94)	6202 (18.46)	
Education (%)				<0.001†
*Primary school or below*	22 461 (53.70)	4858 (59.02)	17 603 (52.39)	
*Secondary school*	16 667 (39.85)	2964 (36.01)	13 703 (40.79)	
*College or above*	2701 (6.46)	409 (4.97)	2292 (6.82)	
Residential location (%)				<0.001†
*Urban*	25 537 (61.05)	4716 (57.30)	20 821 (61.97)	
*Rural*	16 292 (38.95)	3515 (42.70)	12 777 (38.03)	
Live alone (%)	4493 (10.74)	1204 (14.63)	3289 (9.79)	<0.001†
Salty dietary preference (%)	7159 (17.11)	1075 (13.06)	6084 (18.11)	<0.001†
Sweet food preference (%)	2365 (5.65)	441 (5.36)	1924 (5.73)	0.194
Whole grain preference (%)	2489 (5.95)	450 (5.47)	2039 (6.07)	0.039†
Preference for fruits/vegetables (%)	4496 (10.75)	844 (10.25)	3652 (10.87)	0.106
Current alcohol consumption (%)	2156 (5.15)	337 (4.09)	1819 (5.41)	<0.001†
PASE score (IQR)	81.7 (46.7–118.1)	85.0 (50.0–128.0)	80.2 (45.0–116.4)	<0.001†

CKM – cardiovascular-kidney-metabolic, IQR – interquartile range, PASE – physical activity scale for the elderly

*P-values were calculated using the Kruskal-Wallis test for continuous variables (Age, PASE score) and the χ^2^ test for categorical variables.

†Statistically significant results.

### Association of PASE and CKM Syndrome

**Table 2** presents the associations between physical activity (PASE) and CKM syndrome across different adjustment models. In the unadjusted mode, higher PASE quartiles were significantly associated with lower odds of CKM syndrome (*P* for trend <0.001). After sequential adjustment for demographic and lifestyle factors, this association was attenuated but remained statistically significant (Models 2 and 3). Across all models, participants in the highest PASE quartile exhibited approximately 20–25% lower odds of CKM syndrome compared with those in the lowest quartile, supporting the hypothesis that higher physical activity levels are independently associated with reduced CKM risk (**Table 2**).

**Table 2:** PASE in relation to CKM

Variable	Model 1*		Model 2*		Model 3*	
	**OR (95% CI)**	***P-*value**	**OR (95% CI)**	***P-*value**	**OR (95% CI)**	***P-*value†**
PASE						
Per IQR increase‡	0.925 (0.905–0.945)	<0.001†	0.928 (0.907–0.949)	<0.001†	0.923 (0.902–0.944)	<0.001†
*Q1*	Ref		Ref		Ref	
*Q2*	1.016 (0.947–1.089)	0.661	1.055 (0.982–1.133)	0.142	1.042 (0.970–1.120)	0.255
*Q3*	0.953 (0.889–1.021)	0.174	0.997 (0.928–1.071)	0.940	0.985 (0.917–1.059)	0.685
*Q4*	0.792 (0.741–0.847)	<0.001†	0.805 (0.750–0.864)	<0.001†	0.791 (0.737–0.849)	<0.001†
*P* for trend	<0.001†		<0.001		<0.001†	

CI – confidence interval, OR – odds ratio, PASE – physical activity scale for the elderly

*Adjusted models: Model 1 (unadjusted); Model 2 (adjusted for sex, age, education, residential location, and living alone); Model 3 (further adjusted for dietary preferences salty food, sweet food, whole grains, and fruits/vegetables, and current alcohol consumption).

†Statistically significant results.

‡The PASE quartile boundaries (Q1 = <46.7; Q2 = 46.7–81.7; Q3 = 81.7–118.1; Q4 = ≥118.1).

### Associations of PASE with CKM syndrome in gender and age subgroups

**Figure 2:**
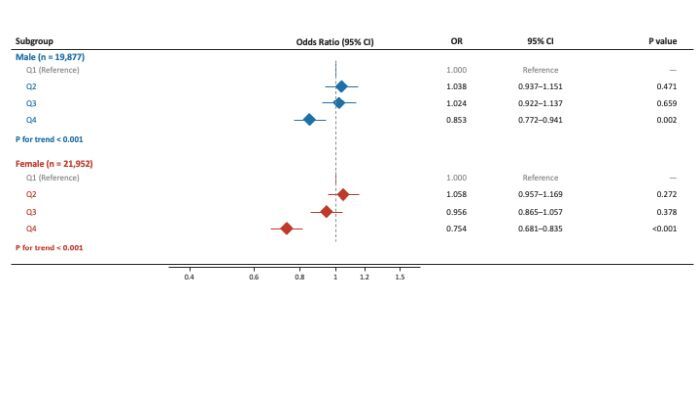
Associations of PASE with CKM in gender-specific subgroups. All models adjusted for sex, age, education, residential location, and living alone, dietary preferences salty food, sweet food, whole grains, and fruits/vegetables, and current alcohol consumption. The PASE quartile boundaries (Q1 = <46.7; Q2 = 46.7–81.7; Q3 = 81.7–118.1; Q4 = ≥118.1). CI – confidence interval, CKM – cardiovascular-kidney-metabolic, OR – odds ratio, PASE – physical activity scale for the elderly.

Gender-subgroup analyses were conducted to examine whether the associations between PASE scores and CKM syndrome differed by gender. The inverse association between physical activity and CKM syndrome remained consistent in both men and women (**Figure 2**). Higher total PASE scores were associated with lower odds of CKM syndrome across increasing quartiles, and the trend persisted after full adjustment for demographic and lifestyle covariates (*P* for trend <0.01 for both sexes).

**Figure 3:**
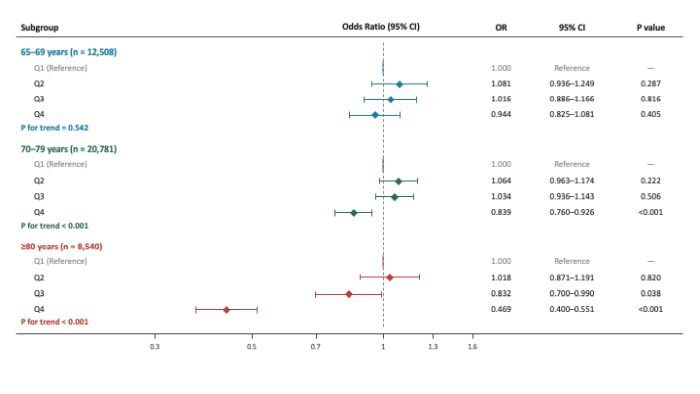
Associations of PASE with CKM in age-specific subgroups. All models adjusted for sex, age, education, residential location, and living alone, dietary preferences salty food, sweet food, whole grains, and fruits/vegetables, and current alcohol consumption. The PASE quartile boundaries (Q1 = <46.7; Q2 = 46.7–81.7; Q3 = 81.7–118.1; Q4 = ≥118.1). CI – confidence interval, CKM – cardiovascular-kidney-metabolic, OR – odds ratio, PASE – physical activity scale for the elderly.

Age-subgroup analyses were further performed to explore potential effect modification by age groups (65–69, 70–79, and ≥80 years). The inverse association between PASE scores and CKM syndrome varied across age strata (**Figure 3**). Among participants aged ≥80 years, higher PASE scores were significantly associated with lower odds of CKM syndrome, and the trend remained robust after full adjustment (*P* for trend <0.001). In contrast, the associations were nonsignificant among those aged 65 to 69 years. These findings suggest that maintaining higher levels of physical activity, particularly in very old adults, may be more strongly associated with lower odds of CKM syndrome (**Figure 3**).

To formally confirm this age-dependent pattern, a multiplicative interaction term (PASE × age group) was evaluated in the fully adjusted model. The likelihood ratio test revealed a highly significant interaction (LR χ^2^ = 27.607, df = 2, *P* for interaction <0.001). This formally demonstrates that the inverse association between physical activity and CKM syndrome genuinely strengthens with advancing age, rather than merely reflecting differential statistical power across the age strata (**Table 3**).

**Table 3:** Formal test of effect modification by age group: PASE × age group interaction in the association between PASE and CKM syn*

Age group/term		N		OR (95% CI)		*P-*value
Association between PASE (per IQR increase) and CKM syndrome: stratum-specific ORs						
*65–69 y*		12 508		1.001 (0.944–1.062)		0.965
*70–79 y*		20 781		0.937 (0.895–0.982)		**0.007†**
*≥80 y*		8540		0.774 (0.716–0.836)		**<0.001†**
Interaction terms (reference: age 65–69 y)						
*PASE × age 70–79 y*		-		0.936 (0.869–1.009)		0.085
*PASE × age ≥80 y*		-		0.773 (0.701–0.851)		**<0.001†**

CI – confidence interval, CKM – cardiovascular-kidney-metabolic, IQR – interquartile range, LR – likelihood ratio, OR – odds ratio, PASE – physical activity scale for the elderly

*Likelihood ratio test for PASE × age group interaction: LR χ^2^ = 27.607, df = 2, *P* for interaction <0.001. Stratum-specific ORs represent the change in odds of CKM syndrome per IQR increase in PASE within each age stratum, derived by the delta method from the interaction model. The interaction model was fully adjusted for sex, age (continuous), education, residential location (urban/rural), living alone, dietary preferences (salty food, sweet food, whole grains, fruits/vegetables), and current alcohol consumption. Age group 65–69 y was the reference for interaction terms. The likelihood ratio test compares the model with interaction terms against the model without (Δdf = 2).

†Statistically significant results.

### Sensitivity analyses

Several sensitivity analyses were performed to confirm the robustness of our primary findings. First, multinomial logistic regression modelling each CKM stage separately revealed that the inverse association with physical activity was most pronounced for Stage 4 (OR = 0.867, *P* < 0.001) and Stage 2 (OR = 0.958, *P* = 0.017). Conversely, Stage 1 showed a modest positive association (OR = 1.047, *P* = 0.016), likely reflecting preserved muscle mass in active individuals rather than pathological adiposity (Table S1 in the **Online Supplementary Document**). Second, additionally adjusting for smoking status did not materially alter the primary inverse association (Table S2 in the **Online Supplementary Document**). Third, when redefining Stage 1 with alternative BMI, the results remained directionally consistent. The inverse association was robust using the BMI≥24 kg/m^2^ threshold but was attenuated when applying the more stringent BMI≥28 kg/m^2^ criteria, primarily due to the substantial reduction in the CKM case count and consequent loss of statistical power (Table S3 in the **Online Supplementary Document**). Fourth, to minimise the impact of extreme outliers and reverse causation, we re-analysed the fully adjusted models after excluding participants with PASE scores in the bottom 10th percentile, those aged over 100 years, and those with established CKM Stage 4. Across all these exclusion scenarios, the significant inverse dose-response relationship between PASE and CKM syndrome remained robust (Table S4 in the **Online Supplementary Document**). Finally, restricted cubic spline analysis confirmed a statistically significant nonlinear dose-response relationship between physical activity and CKM syndrome (Figure S1 in the **Online Supplementary Document**). The odds of CKM declined progressively with increasing PASE scores, reaching a nadir at a PASE score of approximately 129, beyond which the confidence intervals widened due to limited data density at extreme activity levels.

## DISCUSSION

In this large, nationwide study of Chinese older adults, higher physical activity was significantly associated with lower odds of CKM syndrome, with a clear trend observed across activity quartiles. Most notably, this inverse association was strongly age-dependent, it was most pronounced in the oldest-old (aged ≥80 years) but was non-significant among their younger counterparts (aged 65–69 years). The association did not significantly differ by sex.

Our finding of an inverse association between physical activity and CKM syndrome aligns with extensive evidence on the benefits of exercise for cardiometabolic health [20,21]. However, our primary contribution is applying the novel, integrated CKM system to a large, non-Western older population, moving beyond the study of single disease components. While our results corroborate emerging findings from other CKM cohorts [2,22], our use of the geriatric-specific PASE tool provides more nuanced evidence for older adults and sets the stage for notable age-dependent pattern in the PASE-CKM association that merits careful interpretation.

Our general finding that higher physical activity is associated with lower CKM risk is strongly supported by well-established biological mechanisms. Physical activity acts as a multi-system modulator that directly counteracts the core pathophysiology of CKM syndrome. Specifically, regular exercise enhances insulin sensitivity, thereby improving glycaemic control and mitigating a primary driver of type 2 diabetes [23,24]. It also reduces chronic low-grade inflammation by lowering levels of pro-inflammatory cytokines like TNF-α and CRP, which are central to the development of metabolic dysfunction and atherosclerosis [25,26]. Furthermore, physical activity improves vascular endothelial function, promotes vasodilation, and helps regulate blood pressure, protecting both cardiovascular and renal systems from damage [27–29]. Thus, the inverse association we observed is consistent with the expected epidemiological manifestation of these synergistic, multi-organ benefits. That said, given the cross-sectional design of this study, these pathways represent plausible, not established, explanations for the observed associations; prospective and interventional studies are needed to confirm causal direction.

The most striking discovery of our study is this profound age-dependency. While the significant statistical interaction formally confirms this gradient across age strata, we must be cautious in interpreting this purely biologically in a cross-sectional setting. Biologically, physical activity might help the oldest-old by mitigating age-related muscle loss and improving vascular function [30,31]. However, reverse causation is highly probable in this context. For individuals aged ≥80 years, a low PASE score may primarily serve as a proxy for underlying frailty, significant functional disability, or severe pre-existing disease that restricts mobility. Furthermore, those who survive to 80 years and maintain high activity levels likely represent a ‘healthy survivor’ phenotype with inherently better baseline health [32]. Therefore, the strong inverse association observed in this group likely captures a mix of preserved physical capacity and overall health status, rather than just the independent effect of exercise [33]. In contrast, the non-significant association in the 65–69 age group is likely attributable to a ‘floor effect’, where the low prevalence of CKM syndrome diminishes the statistical power. Critically, this age-dependent pattern was formally confirmed by a likelihood ratio test for the PASE × age group multiplicative interaction in the fully adjusted model (LR χ^2^ = 27.607, df = 2, *P* for interaction <0.001), providing statistical evidence that the observed gradient across age strata reflects genuine effect modification rather than differential statistical power alone.

Our findings carry significant clinical and public health implications, particularly for aging societies like China. Overall, they confirm that higher physical activity is a strong behavioural correlate associated with lower holistic risk of CKM syndrome in older adults. The stage-specific multinomial analysis warrants particular attention regarding Stage 1, where higher PASE was paradoxically associated with modestly increased odds (OR = 1.047). This stage is defined solely by BMI≥23 kg/m^2^ in the absence of other metabolic risk factors. Physically active older adults tend to carry greater lean muscle mass, which elevates BMI without representing pathological adiposity; this likely explains the apparent positive association at Stage 1. The clinically more meaningful stages – Stage 2 (metabolic risk factors and/or CKD) and Stage 4 (established CVD/CKD) – consistently showed significant inverse associations with PASE, supporting the primary finding. The RCS analysis further demonstrated that the PASE-CKM dose-response is nonlinear (*P* for nonlinearity <0.001), with the greatest risk reduction concentrated in the PASE range of approximately 80–130, indicating that even moderate increases in physical activity may confer clinically meaningful cardiometabolic benefit. More importantly, the strong inverse association observed in the oldest-old challenges the common assumption that it is ‘too late’ to promote activity in advanced age. Our observational findings suggest that promoting physical activity among individuals aged ≥80 years should be a high-priority public health strategy. It represents a low-cost, high-impact opportunity to reduce the burden of CKM syndrome in this most vulnerable population segment. This calls for clear action at both the clinical and community levels. Clinically, counselling on physical activity warrants consideration as a component of routine geriatric care [34,35]. At the community level, there is a clear mandate to create accessible, age-friendly programmes that cater to the very old [36]. Our findings provide observational evidence suggesting that such investments may yield the greatest potential benefits in this demographic group.

The strengths of this study include its large sample size, strong representativeness of the community-dwelling population, and its being the first to systematically examine the relationship between physical activity and CKM multimorbidity in an Asian elderly cohort, accompanied by detailed subgroup analyses. However, several limitations merit careful consideration. First, the cross-sectional design precludes causal inference. Reverse causation is a major concern, particularly among the oldest-old (≥80 years), where low physical activity may largely reflect underlying frailty or advanced disease rather than a primary lifestyle factor. Second, our multivariable models are susceptible to residual confounding. The CAHS data set lacks clinical data on medication use, detailed comorbidity severity, and depressive symptoms. Third, while we adjusted for dietary preferences and alcohol use to provide a conservative estimate, these variables could partially act as mediators on the pathway between lifestyle and CKM, rather than strict confounders. Fourth, relying on BMI≥23 kg/m^2^ to define Stage 1 may misclassify active older adults with high muscle mass, although our sensitivity analyses using alternative BMI thresholds generally supported the main findings. Fifth, physical activity was assessed via self-report (PASE), which inevitably introduces recall bias. Finally, although we utilised a large multi-stage sample, complex survey weighting was not applied in our analyses; thus, the precision of our estimates and strict generalisability to the entire Chinese older population should be interpreted with appropriate caution.

## CONCLUSIONS

In this large, nationwide study of older Chinese adults, higher physical activity was independently associated with a lower prevalence of CKM syndrome. This inverse association was strikingly age-dependent and most pronounced in the oldest-old (aged ≥80 years), though reverse causation in this frail demographic cannot be ruled out. These observational findings suggest that promoting accessible physical activity may serve as a high-priority, low-cost strategy to help manage the CKM burden in aging populations globally.

## Additional material


Online Supplementary Document
