# Supplementary material for: Association between physical activity and cardiovascular-kidney-metabolic syndrome in older Chinese adults: a nationwide, cross-sectional study
Source: J Glob Health. 2026 Apr 30;16:04149. doi: 10.7189/jogh.16.04149 (PMC13129894; doi:10.7189/jogh.16.04149)
Supplement: Online Supplementary Document [file jogh-16-04149-s001.pdf]

Supplement to: Liu Q, Shen J, Zhang Y, Zhang C, Fan G, Wang L, Zhou H, Zhang J, Shi H. Association between physical activity and cardiovascular-kidney-metabolic syndrome in older Chinese adults: a nationwide, cross-sectional study. J Glob Health. 2026;16:04149.

Table S1. Association between PASE (per IQR increase) and Each CKM Stage: Multinomial Logistic Regression (Stage 0 as Reference)

CKM Stage	Tote l (n=41829)		Male (n=19877)		Female (n=21952)		65-69 yr (n=12508)		70-79 yr (n=20781)		≥80 yr (n=8540)	
	**OR (95%*CI*)**	***P* value**	**OR (95%*CI*)**	***P* value**	**OR (95%*CI*)**	***P* value**	**OR (95%*CI*)**	***P value***	**OR (95%*CI*)**	***P value***	**OR (95%*CI*)**	***P value***
**Stage 0**	Ref		Ref		Ref		Ref		Ref		Ref	
**Stage 1**	1.047 (1.009,1.087)	**0.016**	1.065 (1.011,1.121)	**0.017**	1.027 (0.972,1.085)	0.337	1.047 (0.985,1.113)	0.137	1.075 (1.020,1.132)	**0.007**	1.009 (0.893,1.141)	0.885
**Stage 2**	0.958 (0.925,0.992)	**0.017**	0.999 (0.950,1.050)	0.971	0.917 (0.872,0.965)	**0.001**	0.992 (0.936,1.050)	0.775	0.963 (0.917,1.012)	0.142	0.860 (0.764,0.967)	**0.012**
**Stage 3**	0.978 (0.935,1.024)	0.346	1.018 (0.958,1.082)	0.557	0.931 (0.869,0.999)	**0.046**	1.032 (0.905,1.176)	0.640	0.983 (0.923,1.046)	0.583	0.882 (0.803,0.968)	**0.008**
**Stage 4**	0.867 (0.832,0.904)	**<0.001**	0.884 (0.835,0.937)	**<0.001**	0.847 (0.798,0.899)	**<0.001**	0.993 (0.992,1.068)	0.841	0.869 (0.820,0.921)	**<0.001**	0.684 (0.616,0.759)	**<0.001**

Abbreviations: OR, odds ratio; CI, confidence interval; IQR, interquartile range; CKM, cardiovascular-kidney-metabolic syndrome; PASE, Physical Activity Scale for the Elderly.
Note: Multinomial logistic regression was applied because the proportional-odds assumption was violated (parallel lines test $p < 0.001$). All models were fully adjusted for sex, age (continuous), education, residential location (urban/rural), living alone, dietary preferences (salty food, sweet food, whole grains, fruits/vegetables), and current alcohol consumption. Values in bold indicate $p < 0.05$.

Table S2. Association between PASE and CKM Syndrome with Additional Adjustment for Smoking Status

PASE	Tote l (n=41829)		Male (n=19877)		Female (n=21952)		65-69 yr (n=12508)		70-79 yr (n=20781)		⩾80 yr (n=8540)	
	**OR (95%*CI*)**	***P* value**	**OR (95%*CI*)**	***P* value**	**OR (95%*CI*)**	***P* value**	**OR (95%*CI*)**	***P value***	**OR (95%*CI*)**	***P value***	**OR (95%*CI*)**	***P value***
Per IQR increase	0.921 (0.900,0.942)	**<0.001**	0.948 (0.918,0.979)	**0.001**	0.898 (0.869,0.927)	**<0.001**	0.973 (0.933,1.014)	0.191	0.948 (0.918,0.978)	**0.001**	0.778 (0.738,0.819)	**<0.001**
Q1	Ref		Ref		Ref		Ref		Ref		Ref	
Q2	1.041 (0.969,1.118)	0.269	1.039 (0.937,1.151)	0.470	1.050 (0.950,1.161)	0.339	1.087 (0.941,1.255)	0.256	1.081 (0.979,1.194)	0.123	0.975 (0.833,1.143)	0.759
Q3	0.982 (0.913,1.055)	0.612	1.023 (0.921,1.135)	0.672	0.946 (0.856,1.046)	0.278	1.036 (0.903,1.187)	0.617	1.061 (0.960,1.173)	0.248	0.780 (0.655,0.928)	**0.005**
Q4	0.786 (0.732,0.844)	**<0.001**	0.850 (0.770,0.939)	**0.001**	0.737 (0.666,0.815)	**<0.001**	0.954 (0.834,1.092)	0.495	0.853 (0.772,0.941)	**0.002**	0.437 (0.372,0.513)	**<0.001**
P for trend	**<0.001**		**<0.001**		**<0.001**		**<0.001**		**<0.001**		**<0.001**	

Abbreviations: OR, odds ratio; CI, confidence interval; IQR, interquartile range; Q, quartile; CKM, cardiovascular-kidney-metabolic syndrome.

Note: This model includes **smoking status** as an additional covariate beyond the fully adjusted Model 3. All other adjustments are identical: sex, age, education, residential location (urban/rural), living alone, dietary preferences (salty food, sweet food, whole grains, fruits/vegetables), and current alcohol consumption. Values in bold indicate $p < 0.05$.

Table S3. Sensitivity Analysis Using Alternative BMI Thresholds to Define CKM Stage 1

PASE	Stage 1 defined as BMI ≥24 kg/m² (n with CKM = 28,773)		Stage 1 defined as BMI ≥28 kg/m² (n with CKM = 17,118)	
	**OR (95%*CI*)**	***P* value**	**OR (95%*CI*)**	***P* value**
Per IQR increase	0.964 (0.945,0.983)	**<0.001**	0.985 (0.967,1.003)	0.110
Q1	Ref		Ref	
Q2	1.061 (0.999,1.127)	0.054	1.077 (1.018,1.139)	**0.010**
Q3	1.020 (0.960,1.085)	0.520	1.053 (0.995,1.115)	0.075
Q4	0.902 (0.849,0.958)	**0.001**	0.962 (0.909,1.019)	0.191
P for trend	**<0.001**		**0.017**	

Abbreviations: OR, odds ratio; CI, confidence interval; IQR, interquartile range; BMI, body mass index; CKM, cardiovascular-kidney-metabolic syndrome.
Note: In the primary analysis, CKM Stage 1 was defined as BMI ≥23 kg/m² (Asian-specific threshold) without other metabolic risk factors. This table reports results when Stage 1 is redefined using BMI ≥24 kg/m² (Chinese overweight threshold) or BMI ≥28 kg/m² (Chinese obesity threshold). All other stage definitions and model covariates are unchanged (Model 3). Values in bold indicate $p < 0.05$.

Table S4. Associations of PASE with CKM in the sensitivity analyses

PASE	Analysis with data excluded participants with a PASE score below 10%, n=37688		Analysis with data excluding those aged over 100 years, n=41790		Analysis with data excluding participants with CKM stage 4, n=34249	
	**OR (95%*CI*)**	***P* value**	**OR (95%*CI*)**	***P* value**	**OR (95%*CI*)**	***P* value**
Per IQR increase	0.901 (0.878,0.926)	**<0.001**	0.926(0.904,0.949)	**<0.001**	0.945(0.923,0.968)	**0.001**
Q1	Ref		Ref		Ref	
Q2	0.990 (0.908,1.080)	0.825	1.054 (0.977,1.137)	0.175	1.112 (1.033,1.197)	**0.005**
Q3	0.936 (0.858,1.022)	0.140	1.002 (0.928,1.081)	0.966	1.046 (0.972,1.127)	0.230
Q4	0.748 (0.686,0.816)	**<0.001**	0.800 (0.742,0.863)	**<0.001**	0.860 (0.800,0.925)	**<0.001**
P for trend	**<0.001**		**<0.001**		**<0.001**	

Abbreviations: OR, odds ratio; CI, confidence interval; IQR, interquartile range.
Adjusted models: Model adjusted for sex, age, education, residential location, and living alone, dietary preferences salty food, sweet food, whole grains, and fruits/vegetables, and current alcohol consumption.
Values in bold indicate $p < 0.05$.

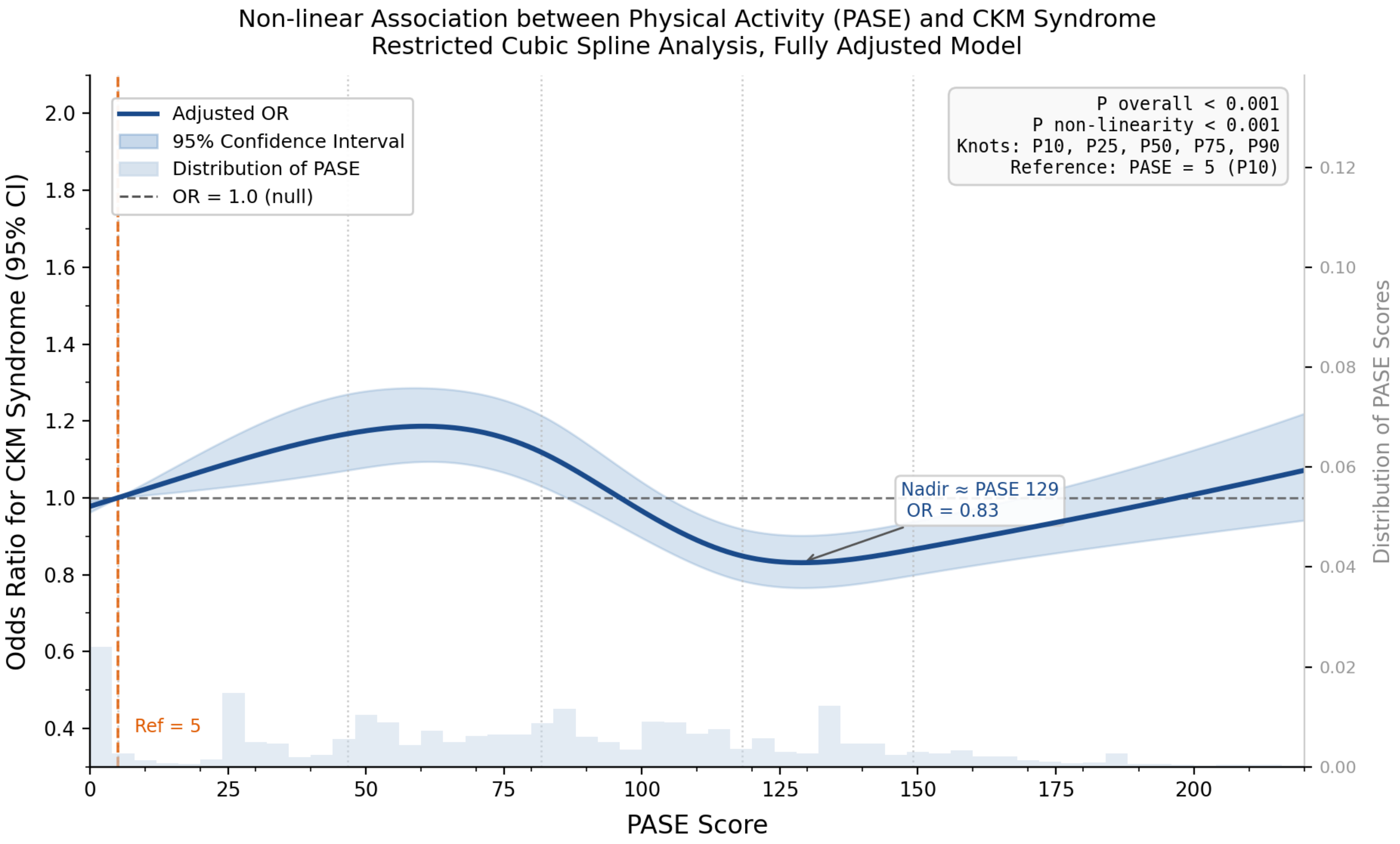

Figure S1. Restricted Cubic Spline Curve for the Association Between PASE and advanced CKM
